# Gene-modified NK-92MI cells expressing a chimeric CD16-BB-ζ or CD64-BB-ζ receptor exhibit enhanced cancer-killing ability in combination with therapeutic antibody

**DOI:** 10.18632/oncotarget.16201

**Published:** 2017-03-15

**Authors:** Ying Chen, Fengtao You, Licui Jiang, Jialu Li, Xuejun Zhu, Yangyi Bao, Xiang Sun, Xiaowen Tang, Huimin Meng, Gangli An, Bozhen Zhang, Lin Yang

**Affiliations:** ^1^ The Cyrus Tang Hematology Center, Soochow University, Suzhou, Jiangsu, PR China; ^2^ Collaborative Innovation Center of Hematology, Soochow University, Suzhou, Jiangsu, PR China; ^3^ Suzhou Cancer Immunotherapy and Diagnosis Engineering Center, Suzhou, China; ^4^ Persongen BioTherapeutics Co., Ltd., Suzhou, Jiangsu, PR China; ^5^ Division of Hematology, Department of Medicine, Jiangsu Provincial Traditional Chinese Medical Hospital, Nanjing, Jiangsu Province, PR China; ^6^ Binhu Hospital, The First People's Hospital of Hefei Group, Hefei, Anhui, PR China; ^7^ Department of Hematology, The First Affiliated Hospital of Soochow University, Suzhou, Jiangsu, PR China

**Keywords:** NK-92MI, chimeric CD16/CD64-BB-ζ receptor, therapeutic antibody

## Abstract

Natural killer (NK) cells play a pivotal role in monoclonal antibody-mediated immunotherapy through the antibody-dependent cell-mediated cytotoxicity (ADCC) mechanism. NK-92MI is an interleukin-2 (IL-2)-independent cell line, which was derived from NK-92 cells with superior cytotoxicity toward a wide range of tumor cells *in vitro* and *in vivo*. Nonetheless, the Fc-receptor (CD16) that usually mediates ADCC is absent in NK-92 and NK-92MI cells. To apply NK-92MI cell-based immunotherapy to cancer treatment, we designed and generated two chimeric receptors in NK-92MI cells that can bind the Fc portion of human immunoglobulins. The construct includes the low-affinity Fc receptor CD16 (158F) or the high-affinity Fc receptor CD64, with the addition of the CD8a extracellular domain, CD28 transmembrane domains, two costimulatory domains (CD28 and 4-1BB), and the signaling domain from CD3ζ. The resulting chimeric receptors, termed CD16-BB-ζ and CD64-BB-ζ, were used to generate modified NK-92MI cells expressing the chimeric receptor, which were named NK-92MI^hCD16^ and NK-92MI^hCD64^ cells, respectively. We found that NK-92MI^hCD16^ and NK-92MI^hCD64^ cells significantly improved cytotoxicity against CD20-positive non-Hodgkin's lymphoma cells in the presence of rituximab. These results suggest that the chimeric receptor-expressing NK-92MI cells may enhance the clinical responses to currently available anticancer monoclonal antibodies.

## INTRODUCTION

Cancer remains the leading challenge among the issues that endanger human health. In the past few decades, cancer incidence and mortality have increased globally. Because the majority of tumors relapse as a result of their resistance to chemotherapy and/or radiation therapy, there is an urgent need to develop curative treatment strategies. In recent years, immunotherapy showed great potential for cancer therapy with several outstanding features, including overcoming chemoresistance and targeting tumor cells selectively. Moreover, immunotherapy has achieved curative effects on various solid tumors and hematological cancers, as suggested by recent data and striking clinical responses. One immunotherapeutic strategy, chimeric antigen receptor (CAR)-modified T-cell therapy, rapidly developed in recent years and showed encouraging anti-tumor efficacy, especially in the treatment of hematological malignancies [1].

The allogeneic NK-92 cell line was derived from peripheral blood mononuclear cells (of a non-Hodgkin's lymphoma patient) with potent cytotoxicity [2]. Therefore, among the efforts to develop an “off-the-shelf” CAR-modified adoptive cell therapy (ACT), research on NK-92 cells expressing CAR produces one of the viable options because they may attain *ex vivo* expansion effectively without labor-intensive steps, while autologous T-cells are needed [3]. Moreover, NK-92 cells can be genetically modified to specifically recognize tumor antigens or to enhance the activity of monoclonal antibodies by antibody-dependent cell-mediated cytotoxicity (ADCC). Indeed, clinical responses with minimal adverse effects have been observed when patients with advanced cancers were infused with NK -92 cells [4]. NK-92MI cells have the same biological characteristics as NK-92 cells do, but can be expanded *ex vivo* without the addition of interleukin-2 (IL-2). Several pre-clinical researches on CAR-engineered NK cells and NK-92 cells have been conducted, and CAR-targeted antigens include CD19, CD20, CD244 ganglioside GD2, CD138, CS1, GPA7, and HER2 [5, 6]. Recently, two clinical studies of CAR-modified NK cells have been initiated (NCT00995137 and NCT01974479). Certainly, NK cells have shown encouraging prospect for adoptive cellular immunotherapy, especially when the CAR-engineered NK cells can further augment their anti-tumor activity with tumor antigen specificity [7–9].

For many tumors, therapeutic antibodies have been utilized widely as a treatment strategy in the last decade [10]. These antibodies include the anti-CD20 monoclonal antibody (mAb) for lymphoma, anti-HER2/neu mAb (trastuzumab) for breast cancer, anti-EGFR mAb (panitumumab) for colorectal carcinoma (CRC), anti-VEGF mAb (bevacizumab) for non-small cell lung cancer (NSCLC), malignant gliomas, and renal cancer, anti-CTLA4 mAb (ipilimumab) for melanoma, anti-CD52 mAb (alemtuzumab) for chronic lymphocytic leukemia (CLL), anti-CD30 mAb (brentuximab vedotin) for Hodgkin's lymphoma, and anti-CD33 mAb (Mylotarg) for acute myeloid leukemia (AML) [11–13]. Biological activities of antibodies depend on the interaction of their Fc region with Fc receptors [14].

Currently, the efficacy of most antibody-based immunotherapeutic strategies largely depends on the recruitment and activation of immune effector cells in the tumor loci [15]. These therapeutic antibodies can target tumor-associated antigens and kill tumor cells by Fc-mediated machineries, including ADCC and ADCP (antibody-dependent cell-mediated phagocytosis) [10, 16]. The ADCC machinery includes the antibody constant fragments (Fcs) binding to a low-affinity Fc receptor that is expressed on the surface of NK cells, such as FcγRIII (CD16), then the antibodies opsonize the targets and drive destruction of targets by NK cells [10, 14]. ADCC has been proven as the major mechanism of the innate immune system against antigen-expressing cancer cells [16, 17], and the main anti-tumor effect of therapeutic antibodies is predominately mediated by NK cells, which express FcγRIII (CD16) [18, 19]. In the meantime, some effector cells, such as macrophages, dendritic cells (DCs), neutrophils, and eosinophils, express a high-affinity Fc receptor, such as FcγRI (CD64), and may cause the destruction of tumor cells via ADCP [16, 20]. Both ADCP and ADCC perform pivotal functions for innate immune cells in response to treatment with a therapeutic antibody [10].

Nevertheless, neither NK-92 nor NK-92MI cells express activating FcγR, and are therefore unable to trigger ADCC [4]. There are a few research groups who tried and successfully expressed CD16 in NK-92 cells [21, 22], although without comprehensive analysis of biological function. We hypothesized that NK-92 or NK-92MI cells with exogenously expressed FcγRs and T-cell–signaling molecules can exert enhanced anti-tumor activity in combination with therapeutic antibodies through ADCC or ADCP [16]. Our initial experimental model designed to examine the efficacy of CD16-BB-ζ and CD64-BB-ζ receptor in NK-92MI cells (referred to as NK-92MI^hCD16^ or NK-92MI^hCD64^ in the text below; hCD16 denotes humanized CD16, and hCD64 means humanized CD64) was CD20-positive non-Hodgkin's lymphoma (NHL). As a heterogeneous class of lymphoproliferative cancers, although most of late stage NHL patients can be effectively treated with high doses of chemotherapeutic drugs, these patients are at a high risk of relapse due to drug resistance, including patients with mantle cell lymphoma (MCL), a distinct subtype of B-cell NHL [23–25]. Accordingly, we tested a novel strategy to combine the anti-tumor effects of NK-92MI cells with an anti-CD20 therapeutic antibody called rituximab to treat MCL in an *in vivo* animal model. We hypothesized that immune effector cells equipped with a CAR, composed of FcγR and T-cell–signaling molecules, would exert ADCC or ADCP activity in combination with the antibody. In this study, we successfully generated gene-modified NK-92MI cells expressing receptor of CD16-BB-ζ or CD64-BB-ζ and demonstrated the possible benefits of this novel therapeutic strategy.

## RESULTS

### Functional validation and characterization of NK-92MI^hCD16^ and NK-92MI^hCD64^ cells *in vitro*

**Figure 1:**
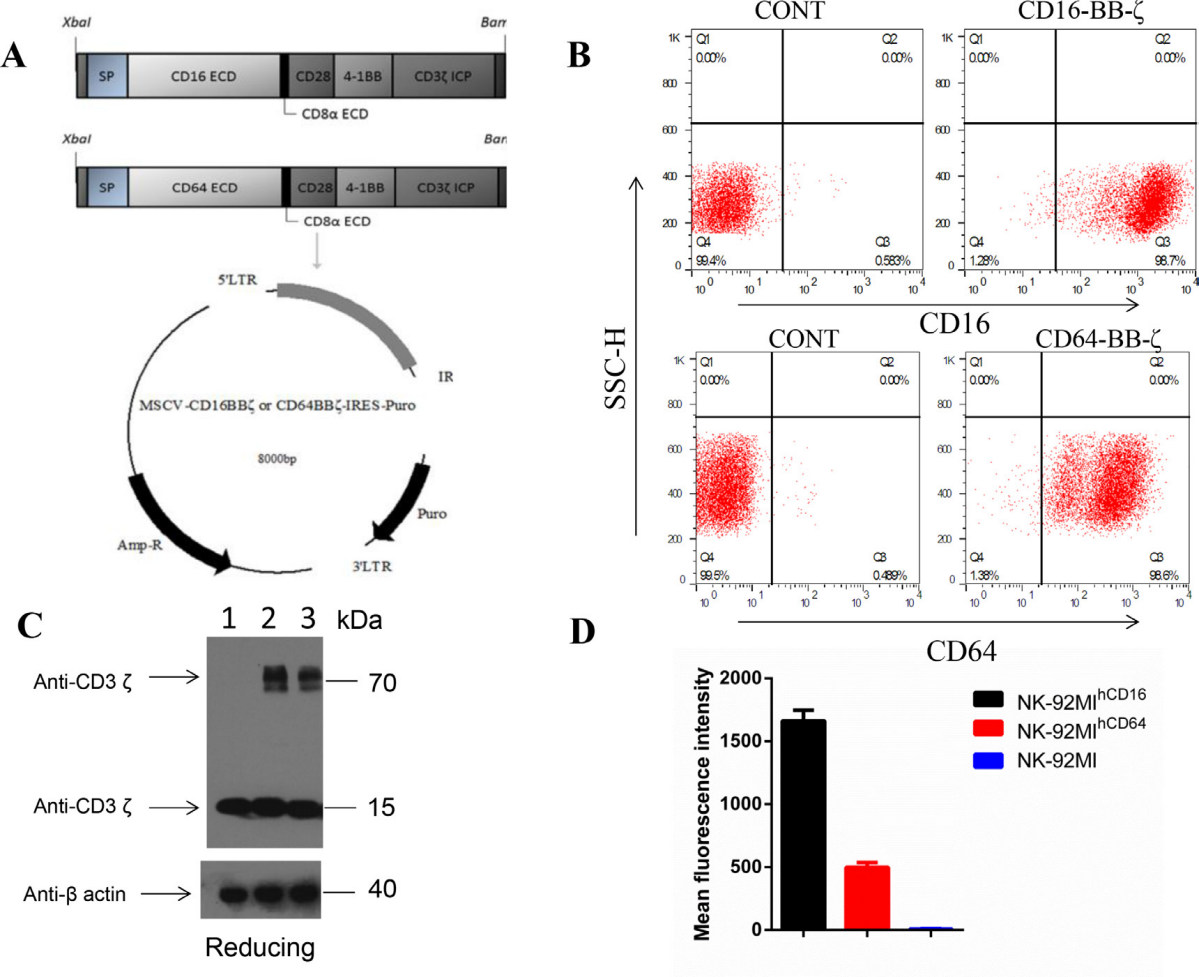
Functional validation and characterization of NK-92MI^hCD16^ and NK-92MI^hCD64^ cells *in vitro* (**A**) Schematic representation of the CD16-BB-ζ and the CD64-BB-ζ receptor constructs. The NK-92MI^hCD16^ and NK-92MI^hCD64^ constructs contained a tandem signaling domain that contained signal peptide (SP), CD16 (158F) cDNA or CD64 cDNA, CD8a extracellular domain (ECD), CD28 transmembrane domain (TM), two co-stimulatory domains (CD28 and 4-1BB) that defined the construct as a “third generation” CAR, and a CD3ζ intracellular signaling domain (ICP). (**B**) Exogenous CD16 or CD64 expression on surfaces of NK-92MI cells are shown. NK-92MI, NK-92MI^hCD16^ and NK-92MI^hCD64^ cells were harvested, stained with anti-CD16 or anti-CD64, and analyzed using flow cytometry. The expression proportion and mean fluorescence intensity (MFI) of CD16 or CD64 are shown in B and (**D**), respectively. NK-92MI cells were negative for CD16 and CD64 expression. Data are presented as the mean ± SD of the three separate experiments. (**C**) Immunoblot analysis of CD3ζ fusion protein expression in NK-92MI^hCD16^ or NK-92MI^hCD64^ cells. Lysates of NK-92 (lane 1), NK-92MI^hCD16^ (lane 2), and NK-92MI^hCD64^ cells (lane 3) were separated by SDS-PAGE under reducing (R) conditions. Immunoblot analysis was performed with a CD3ζ chain-specific mAb followed by exposure to an HRP-conjugated anti-mouse antibody and chemiluminescent detection. The positions of endogenous and chimeric CD3ζ fusion proteins and of molecular weight standards (kDa) are indicated. β-Actin was included as a loading control, 42 kDa.

CD16-BB-ζ's modular design consists of a signal peptide (SP), the CD16 (158F) cDNA (GenBank accession NO.: NM_001127593) extracellular domain (ECD), the CD8a extracellular domain (NM_001768), the CD28 transmembrane domain (TM), and tandem CD28 [26] and 4-1BB [27] co-activation domains linked to the CD3ζ signaling domain previously made in our laboratory [28]. CD64-BB-ζ's modular design is similar to that of CD16-BB-ζ, except for the CD64 cDNA (AK291451) being replaced with the CD16 cDNA (Figure 1A) sequence.

NK-92MI cells transfected with CD16-BB-ζ or CD64-BB-ζ receptor lentivirus were sorted by FACS to enrich NK-92MI^hCD16^ cells or NK-92MI^hCD64^ cells, and the positive ratio of both CD16-BB-ζ and CD64-BB-ζ was 98% with addition of puromycin (1μg/ml), as determined by flow cytometry (Figure 1B). Nevertheless, the mean fluorescence intensity for NK-92MI^hCD16^ was much higher (Figure 1D). After FACS isolation of NK-92MI^hCD16^ or NK-92MI^hCD64^ cells and addition of puromycin (1μg/ml) throughout culture, the cells were maintained with stable CD16- or CD64- positive ratios at approximately 98% among NK-92MI cells during expansion for up to three months. Notably, the CD64 protein expressed in NK-92MI^hCD64^ cells was lost more quickly than the CD16 protein expression in NK-92MI^hCD16^ cells. After the selection of NK-92MI, NK-92MI^hCD16^, and NK-92MI^hCD64^ cells, expression of CD16 mRNA and CD64 mRNA was verified by RT-PCR (Supplementary Figure 5).

Expression of the CD16-BB-ζ or CD64-BB-ζ fusion protein was examined by western blotting. Under reducing conditions, endogenous ζ chain was detected as a 16-kDa band in lysates of NK-92MI (lane 1), NK-92MI^hCD16^ (lane 2), and NK-92MI^hCD64^ cells (lane 3). Two additional bands were observed only in NK-92MI^hCD16^ and NK-92MI^hCD64^ cells, and matched the calculated size (75 kDa) of the CD16-BB-ζ or CD64-BB-ζ fusion protein (Figure 1C). β-Actin (42 kDa) was used to normalize the amounts of cell lysates loaded onto lanes.

### Antibody-binding capacity of receptors CD16-BB-ζ and CD64-BB-ζ in NK-92MI cells

**Figure 2:**
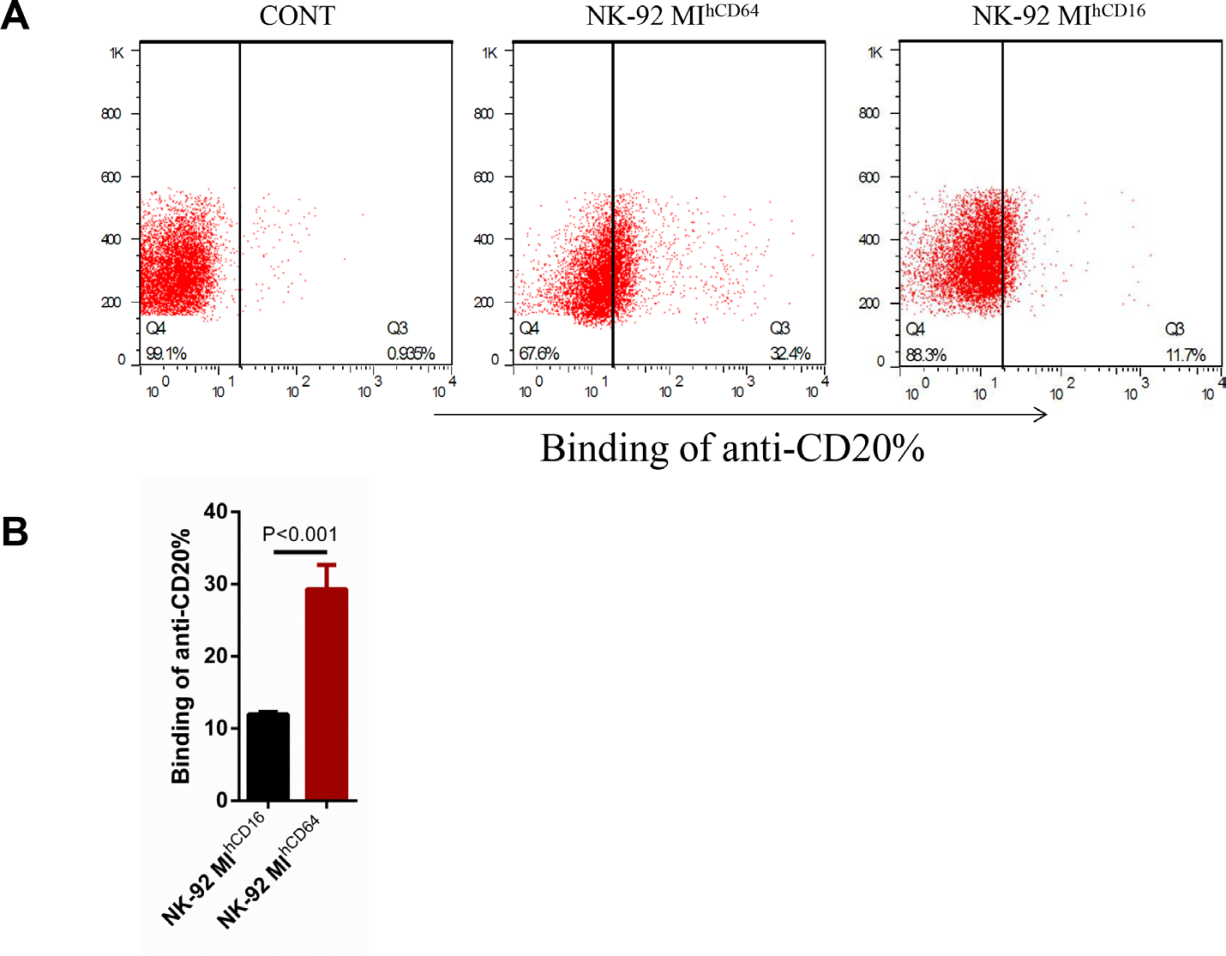
Antibody-binding capacity of CD16-BB-ζ and CD64-BB-ζ receptors in NK-92MI cells (**A**) NK-92MI, NK-92MI^hCD16^, and NK-92MI^hCD64^ cells were incubated with anti-human CD20 antibody for 30 minutes and non-transduced NK-92MI cells were used as a control. The amount of bound antibody was visualized with an anti-human CD20 antibody conjugated to APC by flow cytometry. (**B**) Summary of the antibody-binding capacity assays illustrated in A, (***P < 0.001 by *Student's t-test*). Data are presented as the mean ± SD of three separate experiments.

To compare the binding capacity to rituximab between CD16-BB-ζ and CD64-BB-ζ, we performed an antibody-binding assay in NK-92MI cells. Briefly, NK-92MI, NK-92MI^hCD16^, or NK-92MI^hCD64^ cells were incubated with a human anti-CD20 APC antibody and were analyzed to determine the CD20-positive cell ratio by flow cytometry (Figure 2A). NK-92MI^hCD64^ cells had a much higher APC fluorescence intensity than did NK-92MI^hCD16^ cells, suggesting that NK-92MI^hCD64^ cells had a higher CD20 antibody-binding ability. We also tested the antibody-binding ability of CD16-BB-ζ and CD64-BB-ζ receptors in 293T cells as described above. Similarly, higher APC fluorescence was observed in 293T^hCD64^ cells, indicating that 293T^hCD64^ cells had a higher CD20 antibody-binding ability than 293T^hCD16^ cells did (Supplementary Figure 2).

### NK-92MI^hCD16^ and NK-92MI^hCD64^ cells showed enhanced cytotoxicity in combination with rituximab

Lentivirally gene-modified NK-92MI^hCD16^ and NK-92MI^hCD64^ cells successfully expressed CD16 (98.7% of NK-92MI^hCD16^ cells vs. 0.6% of non-gene modified NK-92MI cells; Figure 1B) and CD64 (98.6% of NK-92MI^hCD64^ cells vs. 0.5% of non-gene modified NK-92MI cells; Figure 1B) on their surfaces respectively.

**Figure 3:**
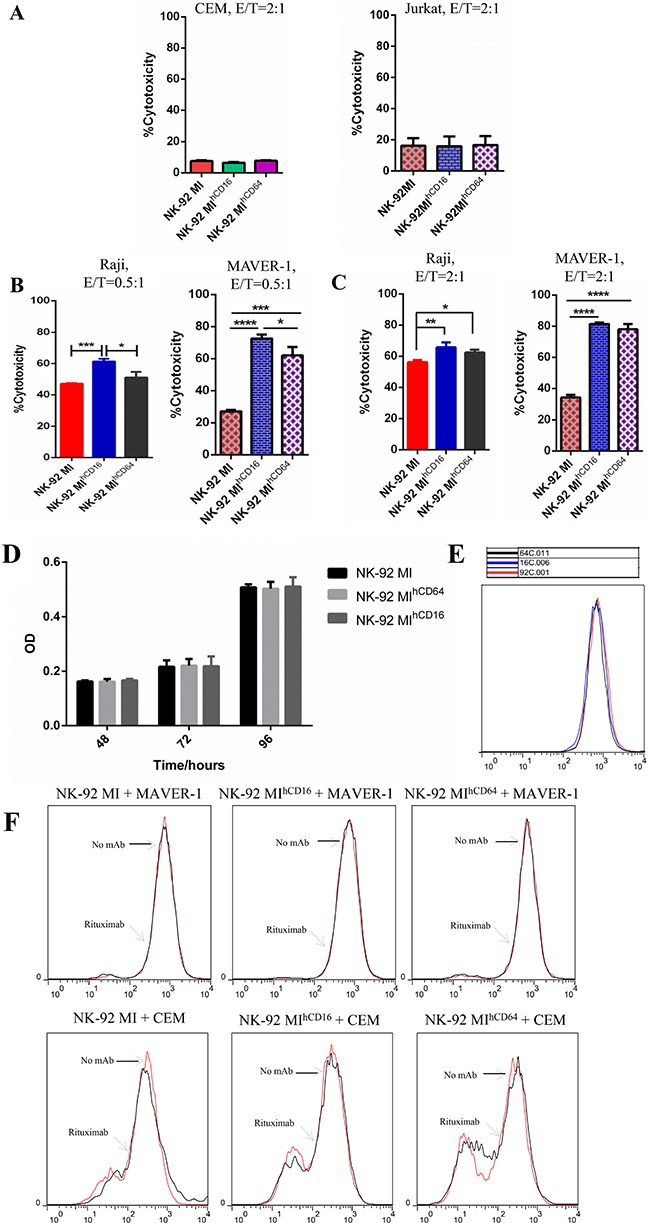
NK-92MI^hCD16^ and NK-92MI^hCD64^ cells showed enhanced cytotoxicity in combination with rituximab (**A**) Spontaneous cytotoxicity of NK-92MI, NK-92MI^hCD16^, and NK-92MI^hCD64^ cells were determined against CEM and Jurkat cells in cytotoxicity assays at an E: T ratio of 2:1. (**B**) Cytotoxicity by NK-92MI, NK-92MI^hCD16^ and NK-92MI^hCD64^ cells against CD20+ cell lines Raji and MAVER-1 in the presence of rituximab at a concentration of 0.1 μg/ml (E:T = 0.5:1). CFSE and 7-AAD assays indicated that NK-92MI^hCD16^ and NK-92MI^hCD64^ cells successfully exerted enhanced cytotoxicity against rituximab-opsonized Raji (left side) and MAVER-1 (right side) cells, but NK-92MI did not exert enhanced cytotoxicity in conjunction with rituximab treatment. (**C**) NK-92MI^hCD16^ and NK-92MI^hCD64^ cells exerted enhanced cytotoxicity against rituximab-opsonized Raji (left side) and MAVER-1 (right side) cells, but NK-92MI did not exert enhanced cytotoxicity when the E:T ratio reached 2:1. Cytotoxicity increased with an increase in E:T ratios. (**D**) Cell proliferation of NK-92MI, NK-92MI^hCD16^, and NK-92MI^hCD64^ cells by MTT assay over time (48 h, 72 h, and 96 h). (**E**) CFSE dilution assay of NK-92MI, NK-92MI^hCD16^, and NK-92MI^hCD64^ cells. (**F**) Proliferative activity in response to rituximab-opsonized MAVER-1 cells or CEM cells mediated by NK-92MI, NK-92MI^hCD16^, and NK-92MI^hCD64^ cells. After ligation with rituximab-opsonized MAVER-1 cells or CEM cells at the indicated dose of rituximab with an E: T ratio of 2:1, NK-92MI, NK-92MI^hCD16^, and NK-92MI^hCD64^ cells did not proliferate. Data are presented as the mean ± SD of three separate experiments. *P < 0.05, **P < 0.01, ***P < 0.001, ****P < 0.0001 compared with NK-92MI at the same E:T ratio. These findings were consistent with the findings of activated NK cells [36].

Prior to evaluating the ADCC effect, we tested whether the overexpression of CD16-BB-ζ or CD64-BB-ζ receptor has any impact on cytotoxicity of NK-92MI cells. The results revealed that there were no significant differences among NK-92MI, NK-92MI^hCD16^, and NK-92MI^hCD64^ cells regarding their ability to kill CEM and Jurkat cells (Figure 3A). Next, the cytotoxicity of NK-92MI, NK-92MI^hCD16^, and NK-92MI^hCD64^ cells against MAVER-1 and Raji cells was examined in the presence of rituximab (0.1 μg/ml) at different E:T ratios. There was no enhanced cytotoxicity observed in the presence of rituximab in NK-92MI cells, while the NK-92MI^hCD16^ and NK-92MI^hCD64^ cells were capable of exerting enhanced cytotoxicity against rituximab-opsonized MAVER-1 cells and Raji cells (Figure 3B and 3C). In contrast, NK-92MI^hCD16^ and NK-92MI^hCD64^ cells could not exert enhanced cytotoxicity against CD138 antibody-opsonized MAVER-1 cells (Supplementary Figure 3).

To minimize the risk of *in vivo* proliferation for the NK-92MI cell line during adoptive immunotherapy, which may result in engraftment and secondary lymphoma in immunocompromised patients, we irradiated the NK-92MI cells, NK-92MI^hCD16^ cells, and NK-92MI^hCD64^ cells at 10 Gy and compared their cytotoxicity with their non-irradiated counterparts. We found that the cytotoxicity of irradiated NK-92MI cells, NK-92MI^hCD16^ cells, or NK-92MI^hCD64^ cells were comparable with cytotoxicity of their non-irradiated counterparts toward CEM, K562, MAVER-1, and Raji cells at the E:T ratios of 2:1 according to flow cytometry (Supplementary Figure 4). Moreover, the proliferative abilities of irradiated NK-92MI cells, NK-92MI^hCD16^ cells, and NK-92MI^hCD64^ cells were completely inhibited when those cells were cultured *in vitro*. Additionally, trypan blue staining revealed that these irradiated NK-92MI cells were nearly dead after cultivation for one week *in vitro*. These findings were consistent with those previously reported [29]. In line with our results, some studies have also shown the non-toxicity and non-carcinogenicity of NK-92 cells [30].

To determine whether the exogenous expression of CD16-BB-ζ or CD64-BB-ζ receptor has any effect on proliferation of NK-92MI cells, MTT and CFSE dilution assays were performed, and the results indicated little or no difference in cell growth rates among NK-92MI, NK-92MI^hCD16^, and NK-92MI^hCD64^ cells *in vitro* (Figure 3D and 3E). We then examined the proliferative activities of NK-92MI, NK-92MI^hCD16^, and NK-92MI^hCD64^ cells when incubated with rituximab-opsonized CD20-positive MAVER-1 cells and CD20-negative CEM cells. As expected, all these cell lines showed similar growth rates (Figure 3F).

### Combination treatment with NK-92MI^hCD16^ cells and rituximab successfully suppressed the growth of a CD20-positive tumor in a xenograft mouse model

**Figure 4:**
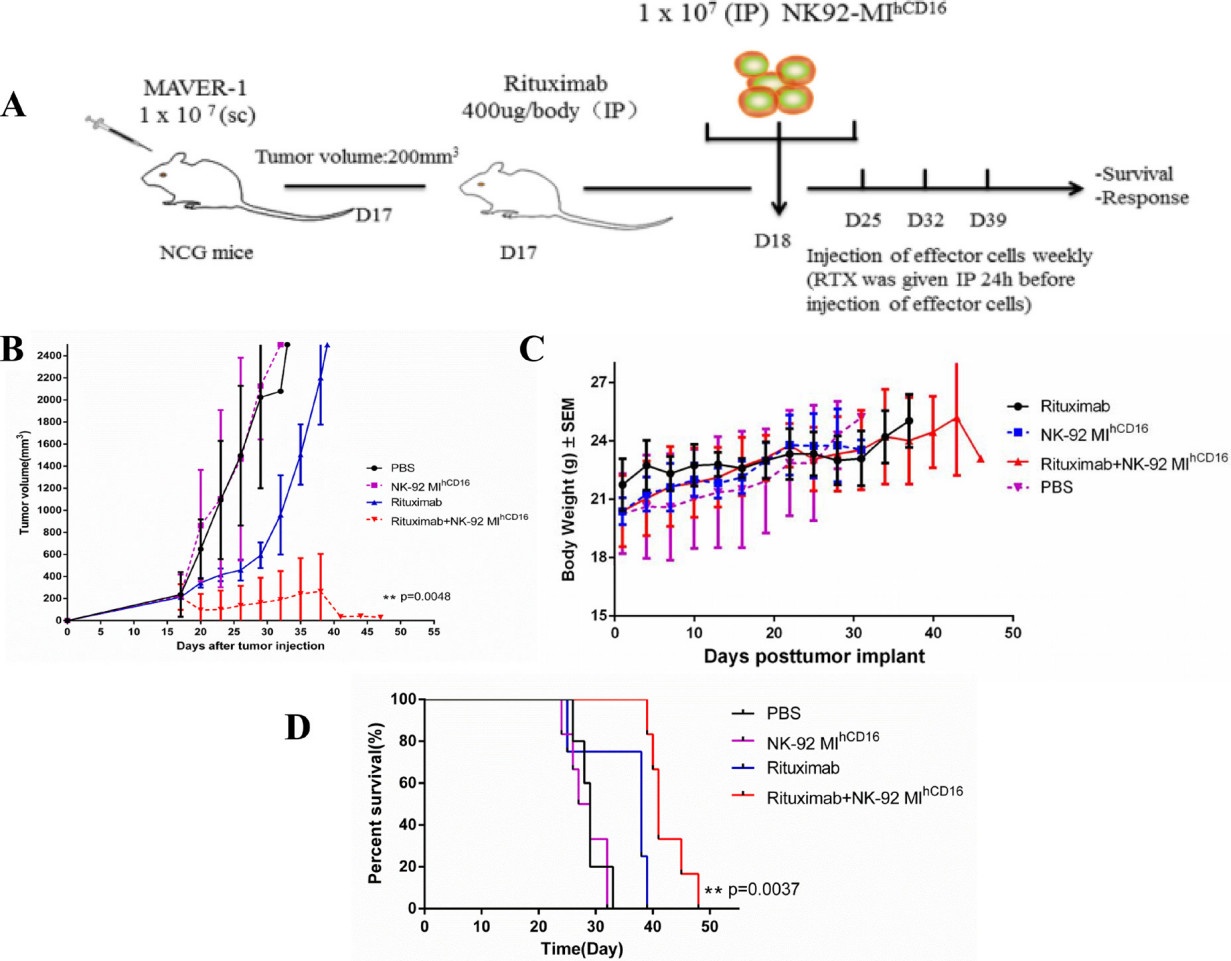
Combination treatment with NK-92MI^hCD16^ cells and rituximab successfully suppressed the growth of a CD20-positive tumor in a xenograft mouse model (**A**) Experiment schema: Seven-week-old female NCG mice were injected subcutaneously in the dorsa; right side with 1 × 10^7^ MAVER-1 cells. When tumors reached a volume of 200 mm^3^, mice were individually identified and randomly assigned to the control or treated groups (4–6 mice per group) and treatments were initiated (all mice were sacrificed before the tumor volume reached 2500 mm^3^). Rituximab (400 μg/mouse) was injected intraperitoneally once weekly for four weeks starting on day 17. Statistical analysis was performed using the log-rank test. (**B**) Tumor volume of the mice was measured every 2–3 days (**P < 0.01). (**C**) Body weights of the mice were measured every 2-3 days. The values are presented as the mean ± standard error of the mean. (**D**) Kaplan–Meier survival curve for mice treated with NK-92MI^hCD16^ cells in combination with rituximab compared to control treated mice. NK-92MI^hCD16^ cells in combination with rituximab treated mice survived significantly longer than the control. Log-rank (Mantel-Cox) test was used to determine significance. (**P < 0.01).

To determine the tumoricidal potential of NK-92MI^hCD16^ cells combined with rituximab in a xenograft model, we evaluated NOD-Prkdc^em26Cd52^ Il2rg^em26Cd22^Nju mice (NCG, Model Animal Research Center of Nanjing University) that received MAVER-1 cells. Seven-week-old female NCG mice were injected subcutaneously on the dorsal right side with 1 × 10^7^ MAVER-1 cells and the mice were administrated with the above-mentioned treatments when the tumor volume reached 200 mm^3^ (mice will be euthanized before the tumor volume reached 2500 mm^3^). In the MAVER-1 xenograft tumor model, NCG mice were injected with NK-92MI^hCD16^ cells and rituximab intraperitoneally four times (Figure 4A). We found that MAVER-1 tumor cell growth in the combination treatment group (NK-92MI^hCD16^ cells and rituximab) was significantly inhibited as compared to mice without any treatment, mice injected with NK-92MI^hCD16^ cells, and mice injected with rituximab only (Figure 4B). This result was also reflected in the survival of the mice. The median survival periods were 33 days for the non-treated group, 32 days for the NK-92MI^hCD16^ group, 39 days for the rituximab group, and 48 days for the NK-92MI^hCD16^ with rituximab group. Furthermore, a statistically significant difference between the NK-92MI^hCD16^ with rituximab group and rituximab-only group was demonstrated (*p* = 0.0037; Figure 4D). Meanwhile, there was no significant decrease in body weight of the mice in all four groups (Figure 4C).

These observations suggest that inoculation with NK-92MI^hCD16^ cells in combination with rituximab suppressed the growth of CD20-positive MAVER-1 cells in the xenograft model. Therefore, we concluded that the combination therapy strategy established in current study was superior in terms of preventing tumor growth comparing with that of NK-92MI^hCD16^ cells or rituximab alone.

## DISCUSSION

Natural killer (NK) cells are a major class of immune effector cells that protect from viral infection and tumor cell invasion in a non-antigen specific manner. Thus, genetically modified CAR-NK cells may acquire novel features, including the ability to specifically recognize tumor antigens with enhanced antitumor cytotoxicity [7]. Other research groups demonstrated the feasibility of expressing cDNA in NK cells by gene transfection [21]. Another study suggests that a patented CD16. NK-92 cell line that expresses either high- or low-affinity CD16 proteins can kill tumor cells *in vitro* when combined with tumor-specific antibodies [22]. Accordingly, we designed and generated two CARs, the low-affinity Fc receptor CD16 (158F) and the high-affinity Fc receptor CD64, with the addition of the CD8a extracellular domain, CD28 transmembrane domains, two costimulatory domains (CD28 and 4-1BB), and the signaling domain from CD3ζ. The rationale for this CAR construction strategy is to obtain a clinically tested NK-92 or NK-92MI cell line that lacks endogenous FcRs and expresses the IgG Fc fragment to give them a less pro-inflammatory profile with simultaneous good effector functions via a variety of machineries, including ADCC or ADCP [31]. Several pre-clinical studies on CAR NK cells and NK-92 cells have been conducted and CAR-targeted antigens include CD19, CD20, CD244 ganglioside GD2, CD138, CS1, GPA7, and HER2 [5, 6]. Recently, two clinical studies of CAR-modified NK cells have begun (NCT00995137 and NCT01974479).

NK-92 cells can exert strong cytotoxicity against a variety of tumors, such as, leukemia, lymphoma, myeloma, and some solid tumors [2, 32, 33]. Some clinical studies have shown the safety of higher doses of NK-92 cell infusion [3]. Unlike CAR modified autologous T-cells, CAR modified NK-92 or NK-92MI cells have several advantages, e.g.: (1) they can kill tumor cells directly with the release of toxic granules, (2) they can augment anti-tumor activity of therapeutic antibodies via ADCC when they were equipped with Fc receptors, (3) they may release a small amount cytokines and can reduce the risk of cytokine storms, and (4) they can be expanded on a large scale and developed as “off-the-shelf ” products [3]. Moreover, just like CAR-T cells, CAR-NK-92 or CAR-NK-92MI cells may circumvent chemoresistance as well. Recently, a series of CAR-NK-92 cell lines were generated, including CD3-CAR-NK-92 for peripheral T-cell lymphomas (PTCLs) [6], EGFR-CAR-NK-92 for breast cancer [34], and CD138 or CS1-CAR-NK-92 for multiple myeloma (MM) [29, 35].

The strategy of applying therapeutic antibodies to cancer treatment has been well established. Those tumor antigen-specific antibodies have shown efficacy against cancers with the help of immune effector cells via Fc-mediated ADCC and ADCP mechanisms [10, 15, 16, 36]. The FcγRIIIa (CD16) gene encodes a low-affinity immunoglobulin Fc receptor, whereas the FcγRI (CD64) gene encodes a high-affinity immunoglobulin Fc receptor [37]. Clinical results with antibody therapies may have limitations for several reasons. Activation of immune effector cells by antibodies depends, in part, on the interaction between Fc domain of monoclonal antibodies and the respective Fc receptor (FcR) expressed on effector cell [38]. We genetically transduced the NK-92MI cells with human receptor CD16-BB-ζ (FcγRIIIa fusion protein) or human receptor CD64-BB-ζ (FcγRI fusion protein) to enable ADCC or ADCP activity in the presence of rituximab, or another therapeutic antibody in other tumor settings. Previously, CD16. NK92 cells have been constructed [21], which are NK-92 cells overexpressing with CD16 (158F). Just as the conventional CAR cells, NK-92MI^hCD16^ cells generated in our present study also carry T-cell–signaling molecules. Because NK-92MI cells lack nearly all the immunoglobulin-like receptors (KIRs), it was unclear whether the T-cell–signaling molecules had any influence on the viability of NK-92MI cells. IL-2 affects the cytotoxicity, proliferation, and viability of CD16. NK92 cells, suggesting that they may have some limitations in clinical applications. In contrast, NK-92MI^hCD16^ and NK-92MI^hCD64^ cells are IL-2 independent and more sensitive to irradiation than CD16. NK92 cells are, although the reason for these phenomenon remains unknown [2]. Proliferation of NK-92MI^hCD16^ cells was inhibited even when the dose of irradiation was low. Furthermore, NK-92MI^hCD16^ cells that produced potent levels of IL-2 may have an advantage for immunotherapy. Both NK-92MI^hCD16^ and CD16. NK92 cells can be produced on a large-scale *in vitro*. The antibody-binding ability of NK-92MI^hCD16^ cells seemed to be weaker; however, it also reduces the risk of ‘off-target’ effects because some tumor antigens are expressed on healthy cells to varying degrees.

Specific cytotoxicity against CD20-positive target cells was demonstrated here for both NK-92MI^hCD16^ and NK-92MI^hCD64^ cells. NK-92MI^hCD16^ cells performed better than NK-92MI^hCD64^ cells, although NK-92MI^hCD64^ cells have a significantly stronger CD20 antibody-binding ability (Figure 2). One explanation for this phenomenon may be that there is higher exogenous CD16-BB-ζ expression in NK-92MI^hCD16^ cells than in NK-92MI^hCD64^ cells. Indeed, although both the CD16-positive and CD64-positive ratios were maintained at ~ 98% among NK-92MI cells in the course of the expansion for up to two to three months, the mean fluorescence intensity of CD16-BB-ζ was always much higher than that of CD64-BB-ζ (Figure 1D). Moreover, we found that the CD64 fusion protein was more easily lost over time. Some effector cells, such as neutrophils and macrophages, can express FcγRI (CD64), which is a high-affinity receptor [16, 20]. Additionally, these effectors may also cause the destruction of tumor cells by ADCP in combination with monoclonal antibodies [39, 40]. CD16 positive immune effector cells, especially for NK cells, constitute the backbone of anti-tumor mechanisms of antibody therapy *in vivo* [18, 19]. Therefore, the ADCC effect may represent a more dominant mechanism for NK-92MI^hCD16^ cells than for NK-92MI^hCD64^ cells.

In summary, we generated two stable cell lines, NK-92MI^hCD16^ and NK-92MI^hCD64^, which hold promise as treatment options for mantle cell lymphoma and other tumor types. NK-92MI^hCD16^ and NK-92MI^hCD64^ cells effectively and selectively killed CD20-positive tumor cells in combination with a clinically approved therapeutic antibody, rituximab, and NK-92MI^hCD16^ cells significantly prolonged the survival of mice in combination with rituximab. Therefore, NK-92MI^hCD16^, and NK-92MI^hCD64^ cells may warrant clinical evaluation against mantle cell lymphoma or other tumor types.

## MATERIALS AND METHODS

### Cell lines

The human T-ALL cell lines CEM and Jurkat, the ery-throleukemic cell line K562, the lymphoblastic cell line Raji, and the *Mantle cell lymphoma cell line MAVER-1* were cultured in RPMI-1640 medium (Thermo Scientific) containing 10% fetal bovine serum (Biowest). HEK293T cells were cultured in DMEM (Thermo Scientific) containing 10% fetal bovine serum. NK-92MI is an interleukin-2 (IL-2)-independent Natural Killer cell line derived from the NK-92 cell line by transfection of the IL-2 gene. The base medium for the NK-92MI cell is Alpha Minimum Essential medium without ribonucleosides and deoxyribonucleosides but with 2 mM L-glutamine and 1.5 g/L sodium bicarbonate. To make the complete growth medium, we added the following components to the base medium: 0.2 mM inositol, 0.1 mM 2-mercaptoethanol, 0.02 mM folic acid, and fetal bovine serum to a final concentration of 12.5%. These cell lines were obtained from ATCC. All cell lines were cultured in a medium with 100U/ml penicillin and 100 μg/ml streptomycin (Gibco) at 37°C in a humidified 5% CO_2_-containing atmosphere.

### CD16-BB-ζ receptor and CD64-BB-ζ receptor construction

The lentiviral vector pCDH-CMV-MCS-EF1-CopPuro (System Biosciences) was used for construction of stable CD16-BB-ζ NK-92MI and CD64-BB-ζ NK-92MI cell lines (referred to as NK-92MI^hCD16^ or NK-92MI^hCD64^, respectively, in the following text). The FcRγIIIa CD16 (158F) cDNA extracellular domain (NM_001127593), FcγRI (CD64) cDNA extracellular domain (AK291451), and the CD8a (NM_001768) extracellular domain were obtained from Origene and synthesized. Signal peptide (SP), CD28 transmembrane domain, two co-stimulatory domains (CD28 and 4-1BB) that define the construct as a “third generation” CAR, and a CD3ζ intracellular signaling domain (ICP) were sub-cloned from a MUC1-specific CAR previously made in our laboratory [28]. These molecules were assembled in various combinations using splicing by overlapping extension by PCR (SOE-PCR). The constructs and expression cassettes were sub-cloned into *Xba*I and *Bam*HI sites of the pCDH-CMV-MCS-EF1-CopPuro vector.

### Lentivirus production and transduction

Preparation of lentiviral supernatant was performed as previously described [28]. NK-92MI cells (1 × 10^6^) were counted and inoculated with lentiviral supernatants (MOI = 30). The transduced cells were incubated at 37°C for 4–6 h, then the supernatants were removed and maintained in Alpha Minimum Essential medium with 12.5% FBS. At 72 h post-transduction, NK-92MI cells were immunostained for CD16 or CD64 and qualitatively observed by fluorescence microscope and analyzed by FACSCalibur (BD Biosciences).

### CD16-BB-ζ or CD64-BB-ζ receptor detection on transduced NK-92MI cells

Transduced NK-92MI cells were analyzed by flow cytometry to determine the CD16-positive or CD64-positive cell ratio. Cells were washed and suspended in phosphate-buffered saline (PBS) and subjected to flow cytometry analysis. NK-92MI^hCD16^ cells and NK-92MI^hCD64^ cells were sorted for subsequent experiments. These sorted cells maintained stable expression when cultured over at least 2 months with continuous addition of puromycin at 1 μg/ml.

### Antibody-binding capacity and CFSE dilution assays

To measure the CD16-BB-ζ or CD64-BB-ζ receptor's antibody-binding capacity, NK-92MI cells, NK-92MI^hCD16^ cells, and NK-92MI^hCD64^ cells were incubated with APC-conjugated anti-human CD20 antibody for 30 minutes at 37°C. After washing twice with PBS, cells were collected and cell staining was measured by FACSCalibur (BD Biosciences). We repeated the antibody binding capacity assay in 293T cells using CD20 staining, as described above.

To measure cell proliferation, 1 × 10^6^ NK-92MI cells, NK-92MI^hCD16^ cells, or NK-92MI^hCD64^ cells were seeded in a 6-well plate (Costar). NK-92MI cells were stained with carboxyfluorescein succinimidyl ester (CFSE), MAVER-1 and CEM cells were labeled with rituximab (at 0.1 mg/ml) or vehicle, for 30 minutes at 37°C. The tumor cells were seeded into the wells at 2:1 E: T ratio with NK-92MI cells. Four days later, the relative proliferation levels (mean fluorescence intensity; MFI) of viable NK-92MI cells after co-culture were measured by flow cytometry.

### MTT assay

The proliferation of NK-92MI, NK-92MI^hCD16^, and NK-92MI^hCD64^ cells *in vitro* was measured using the 3-(4,5-dimethylthiazolyl-2)-2,5-diphenyltetrazolium bromide (MTT) assay (Sigma, St. Louis, MO, USA). NK-92MI, NK-92MI^hCD16^ and NK-92MI^hCD64^ cells were seeded into 96-well plates in a density of 5 × 10^4^ cells/well. After 48, 72, or 96 h of incubation, 20 μL of MTS solution was added to each well and incubated at 37°C for 2 h. The absorbance at 650 nm was recorded as a reference, and subtracted from OD_490_ readings to eliminate nonspecific absorbance. Data from individual experiments are presented as the mean percentage of corrected OD_490_ of triplicate cultures ± SD.

### NK-92MI cell cytotoxicity assay

NK-92MI cell cytotoxicity against tumor cells was evaluated in a 4 h CFSE/7-AAD flow cytometry assay [41]. To determine the cytotoxicity mediated by NK-92MI^hCD16^ cells, NK-92MI^hCD64^ cells, and NK-92MI cells, cytotoxicity was determined by the CFSE/7-AAD flow cytometry assay as previously described [28, 42]. Briefly, 5 × 10^5^ target cells (CEM, K562, Jurkat, MAVER-1, and Raji) were labeled with CFSE for 30 minutes at 37°C and were seeded into round-bottomed 24-well plates. NK-92MI cells were added at various E:T ratios, and co-cultured with target cells for 4 h, with or without the antibodies rituximab or the isotype-matched control antibody CD138 (clone ID:587CT7.3.6.5; made in our laboratory [42]) at 0.1μg/ml. Four hours later, cells were collected, re-suspended in an identical volume of PBS, and 7-AAD (7-aminoactinomycin D; BD) was added. The percentage of specific lysis (CFSE-positive and 7-AAD-positive) was calculated by flow cytometry [28]. In some irradiated experiments, we conducted cytotoxicity assays with a 0.1 μg/ml concentration of rituximab. MAVER-1 and Raji tumor cells highly expressing tumor antigen CD20 were labeled with CFSE. CFSE-labeled MAVER-1 and Raji cells were mixed with NK-92MI cells or irradiated NK-92MI cells (10 Gy), respectively, at a ratio of 2:1 and incubated for 4 h. Non-transduced NK-92MI cells served as the control. After 4 h of co-culture, cells were stained with 7-AAD and the percentage of dead tumor cells (CFSE positive and 7-AAD positive) was determined.

### Prevention of mantle cell lymphoma engraftment in NCG mice

To examine the *in vivo* antitumor effect of NK-92MI^hCD16^ cells, we prepared MAVER-1 cells, whose CD20 expression levels were almost one hundred percent (Supplementary Figure 1). NOD-Prkdc^em26Cd52^ Il2rg^em26Cd22^Nju mice (NCG, Model Animal Research Center of Nanjing University) at 7 weeks of age (*n* = 21) were subcutaneously inoculated with 1 × 10^7^ MAVER-1 cells dorsally on the right side. When tumors reached a volume of 200 mm^3^, mice were individually identified and randomly assigned to control or treatment groups (4–6 mice per group) and treatments were initiated. These mice were divided into four cohorts: (i) non-treated mice (*n* = 5), (ii) mice subjected to intraperitoneal administration of 1 × 10^7^ NK-92MI^hCD16^ cells alone (n=6), (iii) mice subjected to intraperitoneal administration of Rituximab (400 μg/mouse) alone (*n* = 4), and (iiii) mice subjected to intraperitoneal administration of both 1 × 10^7^ NK-92MI^hCD16^ cells and 400 μg/mouse of Rituximab (*n* = 6). Intraperitoneal administration of 1×10^7^ NK-92MI^hCD16^ cells with or without rituximab was done four times, on days 18, 25, 32 and 39.

All *in vivo* mouse experiments were managed in accordance with Soochow University experimental animal management regulations. Tumor growth was monitored twice per week by measuring two perpendicular diameters with calipers. Tumor volume (V) was calculated using the following equation: V= (a^2^ × b)/2, where a is the width of the tumor (small diameter) and b the length of the tumor (large diameter), both in millimeters. Irradiated (10 Gy) NK-92MI^hCD16^ effector cells (1 × 10^7^) were injected IP once per week for four weeks. Rituximab (400 μg/mouse) was given IP 24 h before injection of NK-92MI^hCD16^ cells. Mice were sacrificed before the tumor volume reached 2500 mm^3^.

### Statistics

Survival curves were constructed using the Kaplan–Meier method and statistical analysis of survival was performed using a log-rank (Mantel-Cox) test with *P* < 0.05 considered to be significant. Student's *t*-test was used to compare individual values. Statistical analyses were performed using GraphPad Prism5 software. *P*-values below 0.05 were considered to be statistically significant. Correlation analyses were carried out using the correlation test with a confidence interval of 95%.

## SUPPLEMENTARY MATERIALS FIGURES


