# Supplementary material for: Gene-modified NK-92MI cells expressing a chimeric CD16-BB-ζ or CD64-BB-ζ receptor exhibit enhanced cancer-killing ability in combination with therapeutic antibody
Source: Oncotarget. 2017 Mar 15;8(23):37128–39. doi: 10.18632/oncotarget.16201 (PMC5514896; doi:10.18632/oncotarget.16201)
Supplement: Supplementary file 1 [file oncotarget-08-37128-s001.pdf]

Gene-modified NK-92MI cells expressing a chimeric CD16-BB-ζ or CD64-BB-ζ receptor exhibit enhanced cancer-killing ability in combination with therapeutic antibody

Supplementary Materials

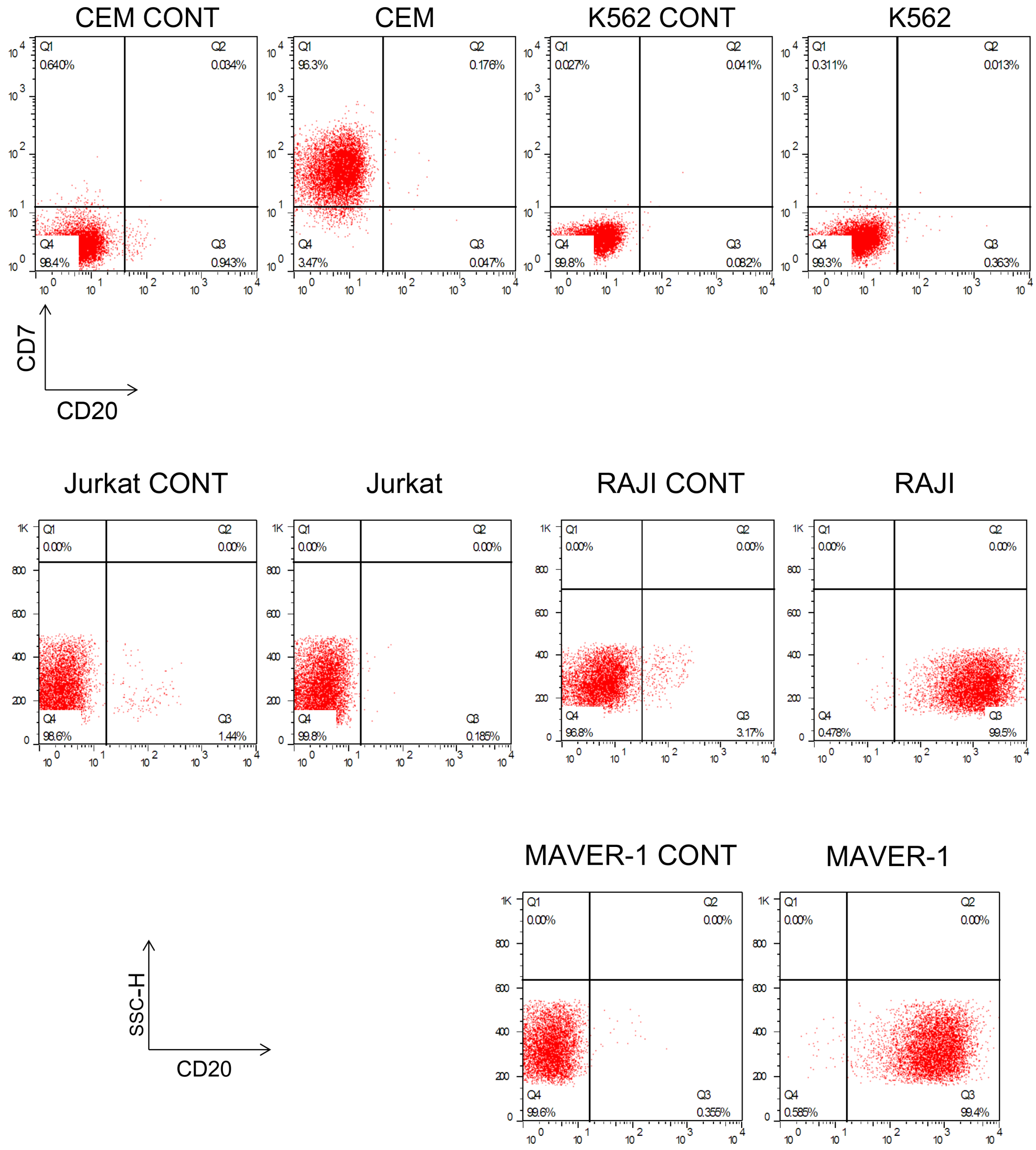

Supplementary Figure 1: Cell line phenotypes. Flow cytometry analysis of T-cell leukemia cell lines CEM and Jurkat, the erythroleukemic cell line K562, the lymphoblastic cell line Raji, and the mantle cell lymphoma MAVER-1. Target cells MAVER-1 and Raji were CD20+ cell line. The CD20+ residuals of MAVER-1 and Raji cell lines existed as a majority population of around 99%. K562 and Jurkat were CD20 negative control cell lines, while CEM was a CD20 negative control cell line that expresses CD7.

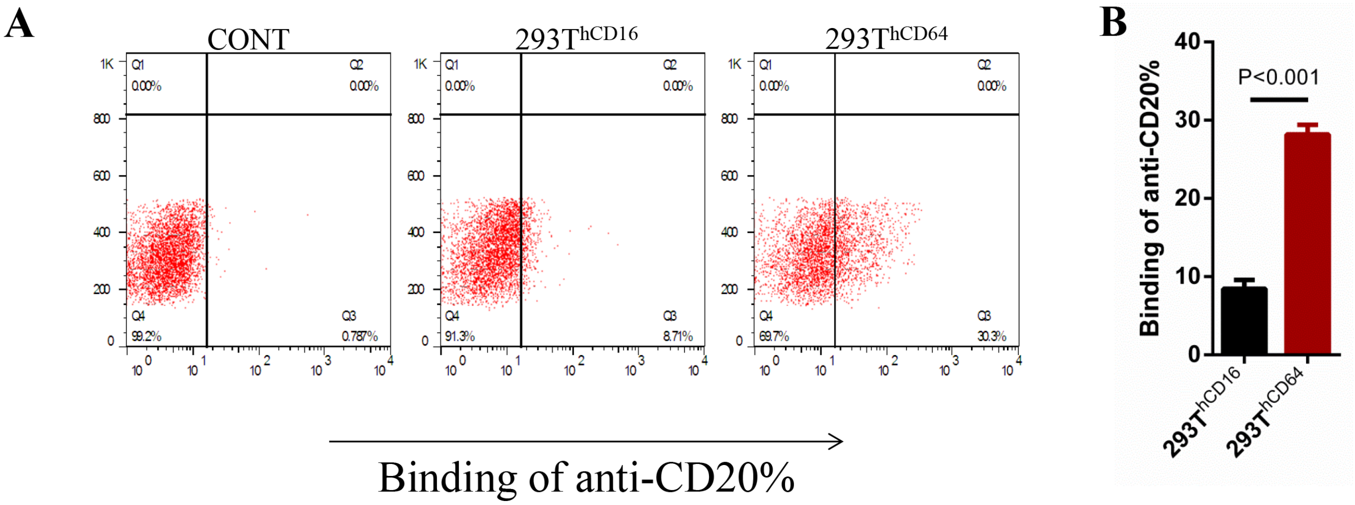

Supplementary Figure 2: Antibody-binding capacity of CD16-BB-ζ and CD64-BB-ζ receptors in 293T cells. (**A**) 293T cells, CD16-BB-ζ 293T cells, and CD64-BB-ζ 293T cells (referred to as 293T^{hCD16} or 293T^{hCD64}) were incubated with human anti-CD20 APC antibody for 30 minutes, non-transduced 293T cells set as control; the amount of antibody bound was visualized with a anti-human CD20 antibody conjugated to APC by flow cytometry assay. CD16 or CD64 expressed nearly 100% in 293T cells (Data not shown). (**B**) Summary of the antibody-binding capacity assays was illustrated in A (***$P < 0.001$ by t test). Data presented are the mean ± SD of three separate experiments.

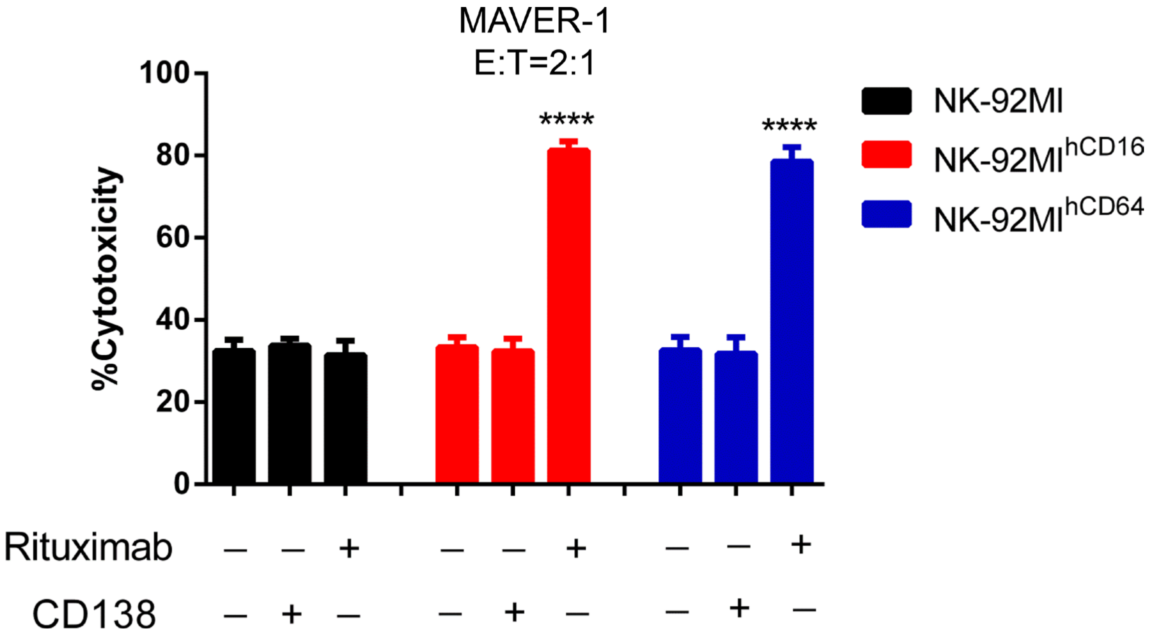

Supplementary Figure 3: Recognition specificity of NK-92MI^{hCD16} and NK-92MI^{hCD64} cells toward target cells expressing CD20. MAVER-1 cells were pre-incubated with 0.1 µg/ml rituximab or an isotype-matched control antibody CD138 (clone ID: 587CT7.3.6.5) which were previously made in our laboratory for 30 min, and then incubated with CFSE for 30 min, followed by co-culture with NK-92MI, NK-92MI^{hCD16} or NK-92MI^{hCD64} cells for 4h. Then, the cytotoxicity of NK-92MI, NK-92MI^{hCD16} or NK-92MI^{hCD64} cells toward these pretreated targets at an E:T ratio of 2:1 were determined by CFSE/7-AAD assay. Data are presented as the mean ± SD of three separate experiments. *$P < 0.05$, **$P < 0.01$, ***$P < 0.001$, ****$P < 0.0001$ compared with the isotype antibody-pretreated group in NK-92MI^{hCD16} or NK-92M^{IhCD64} cells.

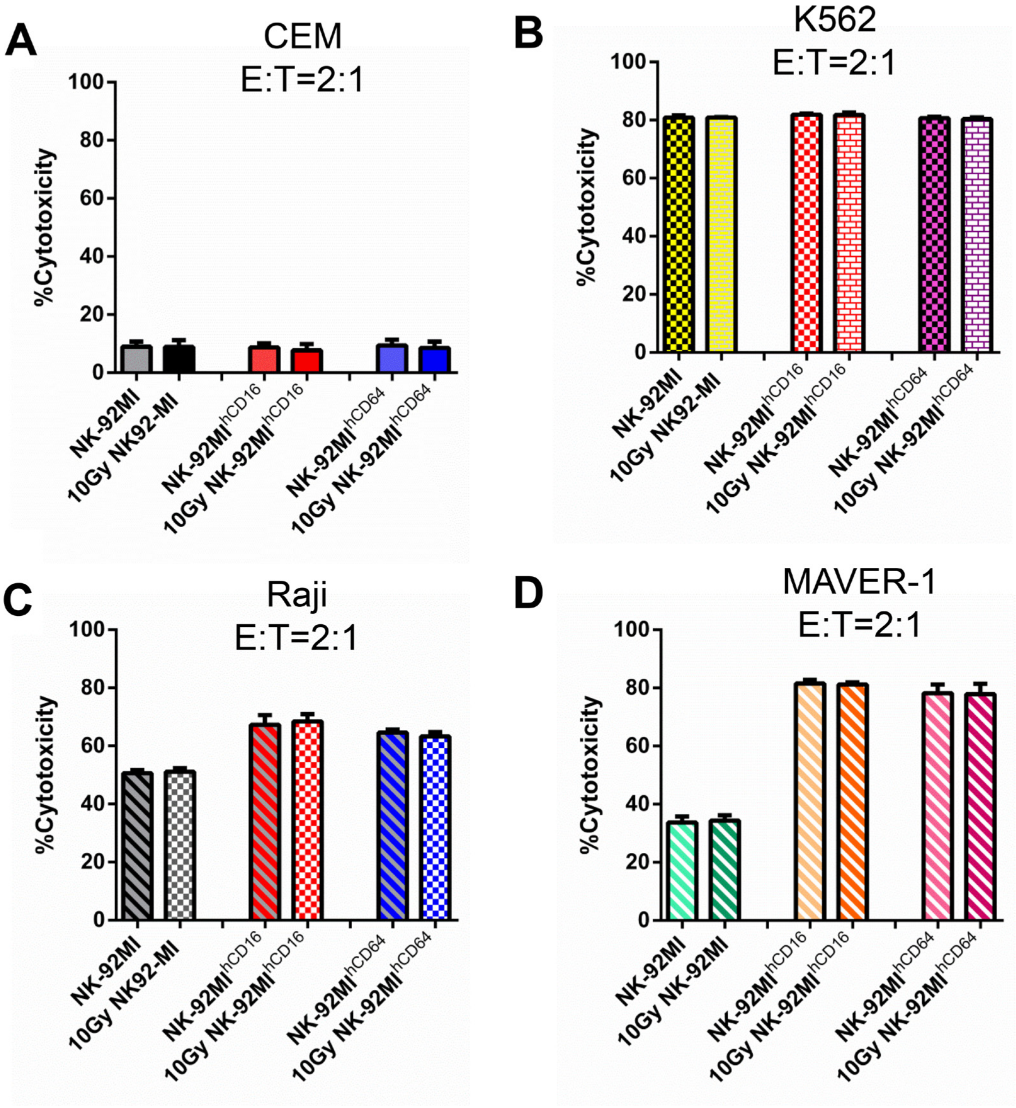

Supplementary Figure 4: Transfected NK-92MI cells retained their cytotoxicity upon irradiation with 10 Gy. T-cell leukemia cell line CEM (**A**), ery-throleukemic cell line K562 (**B**), the lymphoblastic cell line Raji (**C**), and the mantle cell lymphoma cell line MAVER-1 (**D**) were labeled with CFSE and were respectively co-cultured for 4 h with NK-92MI, NK-92MI^{hCD16} or NK-92MI^{hCD64} after irradiation with 10 Gy. Then the cytotoxicity of NK-92MI, NK-92MI^{hCD16} and NK-92MI^{hCD64} towards these tumor cells at E:T ratios of 2:1 were determined by FACS analysis using 7-AAD staining as indicated in materials and methods. Data presented are the mean ± SD of three separate experiments.

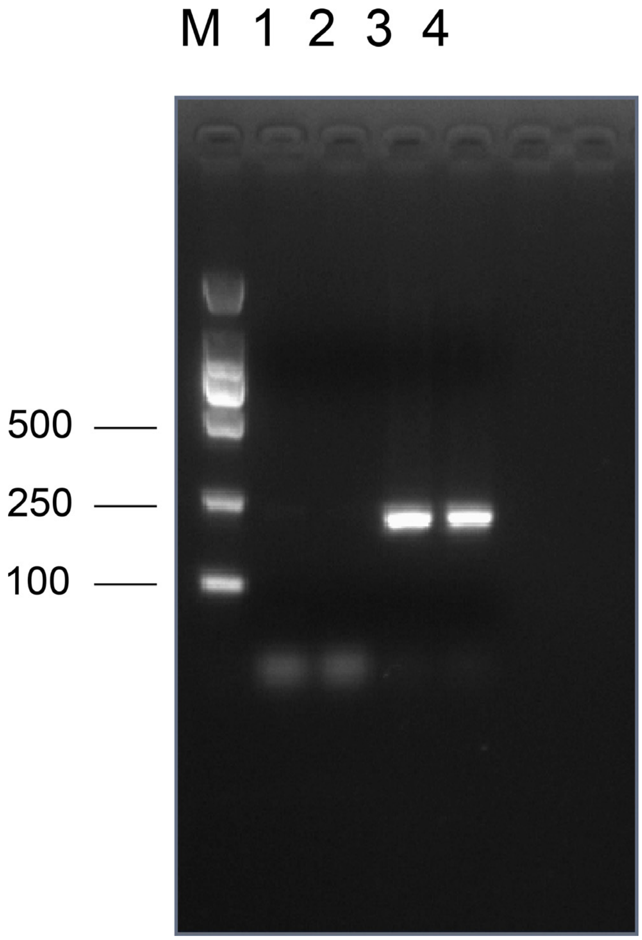

Supplementary Figure 5: The expression of CD16 and CD64 analyzed by RT-PCR. RT-PCR was performed with primers (CD16) specific for the chimeric receptor sequence of mRNA from NK-92MI cells (lane 1), NK-92MI^{hCD16} cells (lane 3), and primers (CD64) specific for the chimeric receptor sequence of mRNA from NK-92MI cells (lane 2), NK-92MI^{hCD64} cells (lane 4). The position of the CD16 (200 bp) and CD64 (200 bp) DNA fragment were indicated. The primers were designed as below: CD16-F: ACTTCATTGACGCTGCCACAGTCGA; CD16-R: TCTGTAAATATGTGACCTTATGCAG; CD64-F: CCCCAGCTACAGAATCACCT CD64-R: TGTACACCAGCTTATCCTTC.
